# Adjunct facet radiofrequency ablation accelerates early recovery trajectories after lumbar Decompression: A prospective matched feasibility study

**DOI:** 10.1016/j.bas.2026.106057

**Published:** 2026-09-05

**Authors:** Pavlina Lenga, Robin Fleige, Max Christian Blumenstock, Matthias Ganzinger, Sebastian Ille, Rezvan Ahmadi, Martin Dugas, Sandro M. Krieg

**Affiliations:** a Department of Neurosurgery, Heidelberg University Hospital, Heidelberg, Germany; b Institute of Medical Informatics, Heidelberg University Hospital, Heidelberg, Germany; c Division of Operative Pain Management, Department of Neurosurgery, Heidelberg University Hospital, Heidelberg, Germany

**Keywords:** Lumbar spinal stenosis, Decompression surgery, Facet joint radiofrequency ablation, Early postoperative recovery, Digital health

## Abstract

**Introduction:**

While lumbar decompression is the standard of care for neurogenic claudication, adequate neural decompression does not address degenerative facet arthrosis, often resulting in residual axial pain that impedes early mobilization. The utility of adjunct facet radiofrequency ablation (RFA) in optimizing the immediate postoperative experience remains under-investigated.

**Research question:**

We therefore hypothesized that adjunct RFA improves early recovery trajectories, observable only through high-frequency monitoring.

**Methods:**

Forty patients undergoing decompression were included; 20 received adjunct bilateral facet RFA based on clinical indication. A post hoc 1:1 matched cohort (age, sex, operated levels) was created for analysis. Early recovery was tracked using a digital well-being scale (0–100) recorded every 48 h for 24 days. Trajectories were quantified using also standard disability (ODI) and mood (PHQ-9) assessments.

**Results:**

Baseline characteristics were comparable (mean age 74.8 vs 75.4 years; baseline ODI 46.7 vs 46.4; baseline PHQ-9 8.8 vs 8.2). Early postoperative well-being was higher in the decompression + RFA group (mean 76.1 ± 9.4) than decompression alone (46.6 ± 11.3), with a paired mean difference of 29.5 points (95% CI, 22.3–36.6; p < 0.001). In mixed-effects trajectory modeling, adjunct RFA was associated with a higher estimated day-0 well-being score (+13.1; p = 0.005) and a steeper recovery slope (group×time +2.49 points/day; p < 0.001). Postoperative ODI was lower with adjunct RFA (26.1 vs 33.4; p = 0.20), while ODI change and PHQ-9 change were similar between groups.

**Discussion and conclusions:**

Adjunct facet RFA significantly optimizes early postoperative well-being and accelerates recovery trajectories following lumbar decompression. By leveraging high-frequency monitoring, this study identifies benefits that standard follow-up intervals fail to capture.

## Highlights

• Adjunct facet RFA enhanced immediate recovery after lumbar decompression.
• High-frequency monitoring revealed recovery gains missed by standard follow-up.
• RFA improved day-0 well-being and accelerated early postoperative trajectories.

## 1 Introduction

Lumbar spinal stenosis (LSS) is a leading cause of pain, walking limitation, and reduced quality of life in older adults and represents a major driver of spine surgery utilization in this age group (Deyo et al., 2010). Decompressive surgery is an established treatment for neurogenic claudication and typically improves pain and function compared with nonoperative care in appropriately selected patients (Weinstein et al., 2010). However, postoperative recovery is heterogeneous, and many patients report persistent symptoms—particularly axial low back pain—despite adequate decompression (Weinstein et al., 2010; Ogon et al., 2022). This residual pain may delay mobilization and negatively influence the early postoperative experience. A key limitation of decompression is that it primarily addresses neural compression but does not directly treat common coexisting pain generators such as degenerative facet arthrosisTo address pain and/or suspected instability, surgeons may add fusion in selected patients (Weinstein et al., 2010; Ogon et al., 2022)., yet randomized evidence shows that fusion does not uniformly improve functional outcomes and increases operative burden, highlighting the need for less invasive adjunct strategies (Försth et al., 2016; Ghogawala et al., 2016). Motion-preserving stabilization approaches exist but introduce implant-related considerations and are not broadly applicable (Musacchio et al., 2016). Radiofrequency ablation (RFA) of the lumbar medial branch nerves (facet denervation) is widely used for facet-mediated low back pain and has a clear anatomic rationale.^16^ In chronic pain populations, evidence is mixed: systematic reviews highlight variability in patient selection and durability (Maas et al., 2008), and sham-controlled trials have questioned long-term effectiveness in unselected cohorts (Maas et al., 2008). Nonetheless, utilization has increased substantially, reflecting ongoing clinical demand for facet-targeted approaches (Manchikanti et al., 2014). Contemporary guidelines emphasize careful selection for facet interventions (Cohen et al., 2020).

In the surgical LSS setting, evidence for adjunct facet denervation is limited. A single-blinded randomized trial found no outcome differences between decompression with versus without open facet denervation at conventional follow-up time points (6–24 weeks) (Faqeeh and Yen, 2019). Importantly, most LSS studies measure outcomes at relatively infrequent intervals (eg, ≥6 weeks), which may miss clinically meaningful differences in the immediate recovery phase (Försth et al., 2016; Faqeeh and Yen, 2019). High-frequency patient-reported monitoring (eg, diaries or mobile tools) is increasingly used in perioperative research to capture recovery trajectories that standard follow-up cannot resolve (Jaensson et al., 2017). Early symptom trajectories after lumbar surgery may also be prognostically relevant, further supporting measurement of the first postoperative weeks with adequate temporal resolution (Sato et al., 2024).

Therefore, we conducted a prospective matched cohort feasibility study comparing decompression with adjunct RFA versus decompression alone in older adults with LSS. We aimed to characterize early recovery using high-frequency postoperative well-being monitoring (single-item 1–100 scale; higher = better, recorded approximately every two days) as an exploratory feasibility endpoint and to contextualize these observations with established patient-reported outcomes including disability (ODI) and depressive symptoms (PHQ-9), thereby generating effect estimates to inform the design of a future prospective randomized trial.

## 2 Methods

### 2.1 Study design

This prospective matched-cohort feasibility study was conducted at the Department of Neurosurgery, Heidelberg University Hospital (UKHD). Data were collected as part of standard clinical practice using an integrated, web-based electronic data capture (EDC) system. The study protocol adhered to the Declaration of Helsinki. Ethical approval for the clinical use of the MyEDC system (S-607/2023) and the scientific analysis of registry data (S-096/2025) was granted by the Ethics Committee of the Medical Faculty, Heidelberg University. All participants provided informed consent for digital data acquisition.

### 2.2 Digital data infrastructure (MyEDC)

Patient-reported outcome measures (PROMs) were collected via "MyEDC," a secure, HTML5-based web application designed for device-independent access. The infrastructure complied with institutional security standards, utilizing a multi-layered firewall architecture and end-to-end encryption. The system automatically dispatched personalized, time-sensitive email invitations containing unique single-use hyperlinks. Upon submission, data were validated and integrated directly into the hospital electronic health record system (i.s.h.med; Cerner Inc.), ensuring real-time clinical availability.

### 2.3 Patient eligibility and matching

Consecutive patients presenting to the outpatient clinic with degenerative lumbar pathology were screened. Inclusion criteria for this analysis were (Deyo et al., 2010): clinically and radiologically confirmed degenerative lumbar spinal stenosis (Weinstein et al., 2010); adult patients (Ogon et al., 2022); scheduled for posterior lumbar decompression (interlaminar fenestration/undercutting); and (Försth et al., 2016) compliance with postoperative high-frequency digital monitoring. To facilitate feasibility analysis and mitigate selection bias in this non-randomized setting, a 1:1 matched cohort was constructed. Patients undergoing decompression plus adjunct radiofrequency ablation (RFA) were matched to those undergoing decompression alone. Matching was performed without replacement using exact matching for sex and operative extent (1 vs. 2 levels), and nearest-neighbor matching for age. This matching strategy was intended to reduce baseline imbalance in selected observed variables but was not designed to eliminate confounding by indication arising from the predefined clinical-radiological phenotype used for RFA selection.

### 2.4 Clinical indication and imaging assessments for adjunct facet radiofrequency ablation

Adjunct facet radiofrequency ablation (RFA) was performed based on a standardized preoperative indication algorithm. In addition to symptoms consistent with lumbar spinal stenosis, patients were considered eligible for adjunct RFA if they presented with predominant axial low back pain provoked by activation/activity (e.g., loading/extension-related pain) and improving after a short walking distance, consistent with a facet-mediated pain phenotype, and if preoperative MRI demonstrated facet joint edema at the index segment(s) (hyperintense signal on T2-weighted fat-suppressed/STIR sequences). The target level(s) for RFA were defined preoperatively and corresponded to the segment(s) with imaging-confirmed facet edema and clinically concordant pain distribution. RFA was performed bilaterally at the treated segment(s) during the same surgical session as decompression. This pragmatic indication algorithm reflected routine clinical care and did not include a separate quantitative rating of axial back pain intensity.

Facet joint edema was assessed on preoperative lumbar MRI (T2-weighted fat-suppressed/STIR sequences). Edema was recorded at each lumbar segment as present/absent at the planned decompression level(s) based on periarticular or intra-articular hyperintensity consistent with active facet joint inflammatory/edematous change. Imaging findings were used to define RFA eligibility and to assign the RFA target level(s).

The decompression-only cohort consisted of patients undergoing decompression during the same clinical period without the standardized RFA indication (i.e., without the combination of activity-related axial back pain improving after a short walking distance and MRI-confirmed facet joint edema at the index level[s]). Accordingly, the comparison reflects real-world routine care in which adjunct RFA is reserved for a defined clinical–radiological phenotype. Matching was applied to reduce baseline imbalance in selected measured characteristics, but the cohorts differ in the preoperative facet-pain phenotype by design; therefore, the comparison should be interpreted as observational and hypothesis-generating rather than causal.

### 2.5 Surgical procedures

Surgical indications were established via multidisciplinary neurosurgical board review based on clinical symptomatology and cross-sectional imaging (MRI/CT). All procedures were performed by board-certified neurosurgeons specializing in spinal surgery.

• Decompression: Standard microsurgical decompression was performed via interlaminar fenestration with targeted undercutting to relieve neural compression while preserving stability.
• Adjunct RFA: In the intervention cohort, RFA of the medial branch nerves was performed bilaterally at the index level(s) under direct visualization, consistent with standard denervation principles.
• Procedural Estimates: For feasibility modeling, operative times were referenced against institutional standards: approximately 60 ± 10 min for single-level and 120 ± 15 min for two-level decompression. Adjunct RFA was estimated to add approximately 20 min per treated level.

### 2.6 Outcome assessments and high-frequency monitoring

Baseline demographic data (age, sex, BMI) were captured preoperatively.

• **High-Frequency Monitoring (Primary Feasibility Endpoint):** To characterize recovery trajectories, a single-item well-being score ("How do you feel today?"; 1–100 visual analog scale) was assessed approximately every 48 h starting immediately postoperatively. This item was used as a pragmatic exploratory digital monitoring measure to capture within-patient recovery kinetics; it is not a formally validated standalone postoperative recovery instrument for this population and may be susceptible to response and reporting bias.
• **Standard PROMs:** Functional disability was assessed using the Oswestry Disability Index (ODI) preoperatively and at follow-up. Depressive symptoms were evaluated using the Patient Health Questionnaire-9 (PHQ-9) at baseline and approximately 6 weeks postoperatively.

### 2.7 Statistical analysis

All analyses were conducted using **R statistical software** (version 4.2.2, R Foundation for Statistical Computing, Vienna, Austria). A 1:1 matched cohort was constructed to compare decompression + RFA versus decompression alone. Matching was performed **without replacement**, using **exact matching on sex** and **operated level category (1 vs 2 levels)** and nearest-neighbor matching on age (minimizing absolute age difference). Analyses were paired by matched set. Post-matching comparability was evaluated descriptively from baseline characteristics (Table 1); no propensity score, caliper restriction, or formal balance target was prespecified for this feasibility study.

**Table 1:** Baseline characteristics (matched cohorts).

Variable	RFA + Decompression	Decompression only	Paired p
Sex (male)	13 (65%)	13 (65%)	
Operated levels (2-level)	10 (50%)	10 (50%)	
Age (years)	74.8 ± 6.9	75.4 ± 5.8	0.124
BMI (kg/m^2^)	27.3 ± 4.9	26.8 ± 3.4	0.475
aCCI	6.5 ± 0.0	6.5 ± 0.0	
Preop ODI (0-100)	46.7 ± 14.3	46.4 ± 14.6	0.961
Baseline PHQ-9 (0-27)	8.8 ± 5.4	8.2 ± 4.9	0.755

Values are mean ± SD or n (%). Paired p-values are shown where applicable.

Continuous outcomes were summarized as mean ± SD. Paired between-group comparisons were performed using **paired t-tests**; **Wilcoxon signed-rank tests** were used as nonparametric sensitivity analyses. Effect sizes for paired comparisons were reported as **Cohen's dz** where applicable. Two-sided p-values <0.05 were considered statistically significant. For high-frequency well-being trajectories, postoperative time was represented in 2-day increments derived from the assessment sequence. Early recovery was quantified in two complementary ways: **AUC-average endpoints** over prespecified early windows (e.g., 0–2 days, 0–4 days, 0–6 days) using trapezoidal integration and normalization to the 1–100 scale; these AUC-average values can be interpreted as the average well-being experienced across the respective early postoperative window, with higher values indicating better overall recovery during that interval. Longitudinal mixed-effects modeling included fixed effects for treatment group, postoperative time, and their interaction, with a random intercept for subject to account for repeated measures. Because of the limited feasibility sample, random slopes were not modeled. A sensitivity model treated time as categorical to avoid assuming linear recovery.

## 3 Results

### 3.1 Cohort and baseline characteristics

Forty patients (20 matched pairs) were analyzed: 20 underwent decompression with adjunct radiofrequency ablation (RFA) and 20 underwent decompression only. Baseline characteristics were were broadly similar with respect to the measured variables shown in Table 1, including age (74.8 ± 6.9 vs 75.4 ± 5.8 years), BMI (27.3 ± 4.9 vs 26.8 ± 3.4 kg/m^2^), baseline ODI (46.7 ± 14.3 vs 46.4 ± 14.6), and baseline PHQ-9 (8.8 ± 5.4 vs 8.2 ± 4.9). Each cohort included 10 single-level (L4/5) and 10 two-level (L4/5 + L5/S1) cases. As intended by the clinical indication algorithm, the RFA cohort represented patients with the predefined facet-pain phenotype, whereas the decompression-only cohort did not.

### 3.2 Surgical characteristics

In the RFA cohort, the first 10 patients underwent bilateral RFA at a single segment (L4/5), and the remaining 10 underwent bilateral RFA at two segments (L4/5 and L5/S1). Estimated decompression times were approximately 62.5 ± 2.8 min for one-level and 122.1 ± 1.2 min for two-level procedures; adjunct RFA required approximately 20 min for bilateral one-level treatment and 40 min for bilateral two-level treatment (Supplementary Table S1).

### 3.3 Primary endpoint – high-frequency postoperative well-being

**Fig. 1:**
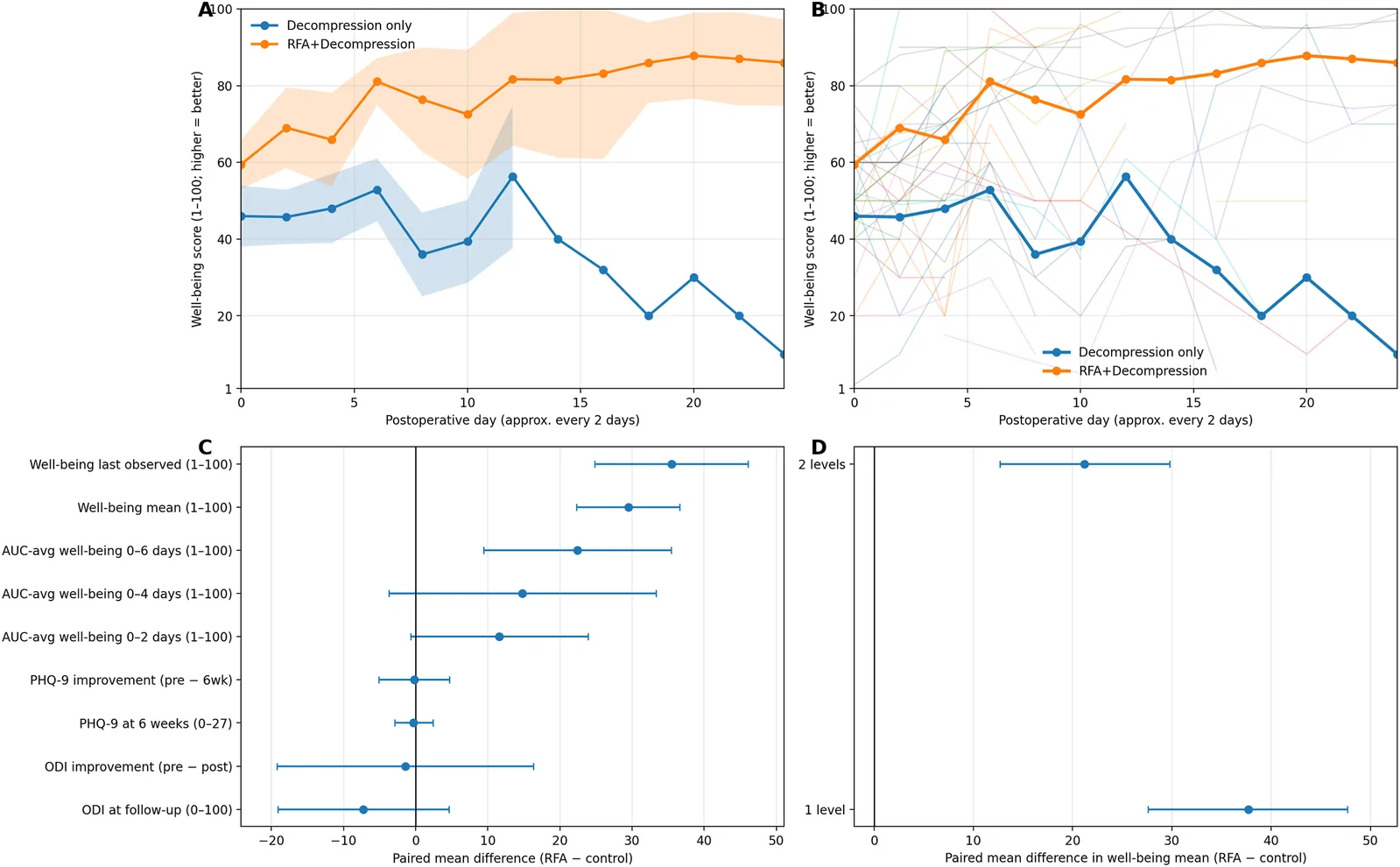
Early postoperative recovery and outcome effects in matched cohorts. (A) Mean postoperative well-being trajectory (1–100; higher = better) with 95% confidence intervals over postoperative days (B) Individual well-being trajectories (thin lines) with cohort means (thick lines). (C) Paired effect estimates (RFA − control) across endpoints with 95% confidence intervals. (D) Paired mean difference in mean well-being by number of decompressed levels (1-level vs 2-level).

Postoperative well-being (score range 1–100; higher = better) was recorded approximately every 2 days and showed sustained separation between cohorts over postoperative days 0–24 (Fig. 1A and B). Mean well-being across monitoring was higher with adjunct RFA (76.1 ± 9.4) than decompression alone (46.6 ± 11.3), corresponding to a paired mean difference of +29.5 points (95% CI 22.3 to 36.6; p < 0.001; Table 2). The last observed well-being score also favored the RFA cohort (79.8 ± 17.4 vs 44.3 ± 21.2; paired difference +35.5 points; 95% CI 24.8 to 46.1; p < 0.001). These between-group differences were observed during the monitored early postoperative period only and should not be extrapolated to mid- or long-term outcomes.

**Table 2:** Clinical outcomes (paired analyses).

Outcome	RFA + Decompression (mean)	Decompression only (mean)	Paired diff (RFA-Ctrl)	95% CI low	95% CI high	p (paired t)	p (Wilcoxon)	Effect size (dz)
Well-being (mean, 1-100)	76.1	46.6	29.5	22.3	36.6	<0.001	<0.001	1.9
Well-being (last observed, 1-100)	79.8	44.3	35.5	24.8	46.1	<0.001	<0.001	1.6
AUC-avg well-being 0-2d	61.6	50.0	11.6	−0.7	23.9	0.062	0.050	0.6
AUC-avg well-being 0-4d	61.7	46.9	14.8	−3.7	33.3	0.101	0.109	0.7
AUC-avg well-being 0-6d	66.2	43.8	22.4	9.4	35.4	0.009	0.062	2.1
ODI at follow-up (0-100)	26.1	33.4	−7.3	−19.1	4.6	0.201	0.203	−0.4
ODI improvement (pre - post)	17.4	18.8	−1.4	−19.2	16.3	0.859	0.846	−0.1
PHQ-9 at 6 weeks (0-27)	3.4	3.6	−0.3	−2.9	2.4	0.825	0.726	−0.1
PHQ-9 improvement (pre - 6wk)	4.9	5.1	−0.2	−5.1	4.7	0.936	0.959	−0.0

Paired difference is RFA minus Control. Effect size is paired Cohen's dz.

### 3.4 Early recovery quantification (AUC endpoints)

Prespecified AUC-average well-being endpoints supported higher early recovery in the RFA cohort (Table 3). Differences favored RFA across early windows, reaching statistical significance for the 0–6 day AUC-average endpoint (66.2 vs 43.8; paired difference +22.4 points; 95% CI 9.4 to 35.4; p = 0.009).

**Table 3:** Early recovery endpoints derived from high-frequency well-being monitoring (AUC-average).

Endpoint	RFA mean	Control mean	Diff (RFA-Ctrl)	95% CI	p (paired t)
AUC-avg well-being 0-2d	61.6	50.0	11.6	−0.7 to 23.9	0.062
AUC-avg well-being 0-4d	61.7	46.9	14.8	−3.7 to 33.3	0.101
AUC-avg well-being 0-6d	66.2	43.8	22.4	9.4 to 35.4	0.009

### 3.5 Trajectory inference (mixed-effects model)

In linear mixed-effects modeling of repeated well-being scores (fixed effects for group, time, and group-by-time interaction; random intercept for subject), adjunct RFA was associated with a higher estimated score at postoperative day 0 (+13.1 points; p = 0.005) and a significantly steeper recovery slope (+2.49 points/day for the group-by-time interaction; p < 0.001), consistent with a faster early improvement trajectory in the matched RFA cohort.

### 3.6 Disability and depressive symptoms

Postoperative ODI at follow-up was numerically lower in the RFA cohort (26.1 vs 33.4), but the paired difference did not reach statistical significance (−7.3 points; 95% CI −19.1 to 4.6; p = 0.201; Table 2). ODI improvement from baseline was similar between cohorts (17.4 vs 18.8; paired difference −1.4 points; 95% CI −19.2 to 16.3; p = 0.859). PHQ-9 at approximately 6 weeks was comparable (3.4 vs 3.6; paired difference −0.3; 95% CI −2.9 to 2.4; p = 0.825), and PHQ-9 improvement from baseline was similar (4.9 vs 5.1; paired difference −0.2; 95% CI −5.1 to 4.7; p = 0.936).

### 3.7 Exploratory subgroup by operated levels

The well-being advantage for RFA was observed in both single-level and two-level surgery strata (Fig. 1D; Supplementary Table S2). For single-level procedures, mean well-being was 83.2 with RFA versus 45.5 with decompression only (paired difference +37.7 points; 95% CI 27.6 to 47.7; p < 0.001). For two-level procedures, mean well-being was 69.0 versus 47.8 (paired difference +21.2 points; 95% CI 12.7 to 29.8; p < 0.001).

### 3.8 Sensitivity analyses and effect sizes

Wilcoxon paired tests were consistent with the primary paired *t*-test results for well-being (Table 2). Standardized paired effect sizes were large for mean well-being (dz = 1.9) and last observed well-being (dz = 1.6), while effect sizes for ODI and PHQ-9 endpoints were small (Fig. 1C).

## 4 Discussion

### 4.1 Principal findings and context

In this prospective matched cohort feasibility study, the use of adjunct radiofrequency ablation (RFA) during lumbar decompression was associated with better early postoperative well-being than decompression alone. Through high-frequency monitoring (every 48 h) using an exploratory single-item 1–100 scale, the RFA cohort reported higher scores throughout the first 3–4 postoperative weeks and a steeper recovery trajectory. These between-group differences were confined to the early monitored period and should not be interpreted as evidence of mid- or long-term benefit, particularly because ODI and PHQ-9 at later follow-up did not differ significantly between groups. While both groups achieved expected improvements in stenosis-related symptoms and functional status, the matched observational design does not permit attribution of the observed differences to RFA alone, because treatment allocation was based on a predefined clinical-radiological phenotype. Methodologically, this study moves beyond standard cross-sectional outcomes (e.g., ODI, PHQ-9) by utilizing high-frequency well-being monitoring. To our knowledge, high-frequency postoperative well-being monitoring during the first weeks after lumbar decompression has not previously been reported in the spine surgery literature. Conventional follow-up schedules (often beginning at approximately 6 weeks postoperatively) provide limited temporal resolution and therefore do not characterize short-lived dynamics in early convalescence. Building on our group's prior prospective PROM work in degenerative lumbar spine disease at a tertiary neurosurgical center (Lenga et al., 2026), we implemented repeated sampling in the first postoperative month and quantified recovery kinetics using AUC summaries and trajectory modeling, which identified early robust between-group separation that could be missed with conventional follow-up alone.

### 4.2 Comparison with existing literature

Lumbar decompression has proven efficacy for relieving neurogenic claudication and functional impairment in selected patients (Weinstein et al., 2010)[. However, decompression alone does not mitigate axial low back pain arising from degenerative facet arthrosis, a comorbidity that frequently contributes to persistent postoperative symptoms (Ogon et al., 2022; Kim et al., 2013). Reoperation rates following decompression-only procedures—estimated between 10% and 25%—may be partly attributable to residual pain from such segmental pathology (Kim et al., 2013). While invasive strategies (e.g., fusion) or interlaminar stabilization devices can address instability or offload facets, they are associated with increased procedural complexity, cost, and implant-related morbidity (Son et al., 2021). In this context, facet RFA offers a distinct therapeutic strategy: the targeting of facet-mediated nociception without the biomechanical alterations inherent to instrumentation. Outcomes in the pain management literature regarding RFA are mixed. Some placebo-controlled trials indicate only short-term functional improvement without durable pain relief (Maas et al., 2015). while others report benefits persisting for 6–12 months (Ghogawala et al., 2016; Musacchio et al., 2016; Son et al., 2021). However, outcomes in the pain management literature regarding RFA remain mixed. Systematic review data and sham-controlled trials have raised questions regarding durability and the influence of patient selection (Cohen et al., 2020; Maas et al., 2015; Juch et al., 2017). Accordingly, our data should be interpreted within the context of a selected clinical phenotype and an early postoperative time window.

Within the specific population of patients undergoing surgery for lumbar spinal stenosis, evidence on intraoperative or perioperative adjunct facet denervation remains limited. A single-blinded RCT (Faqeeh and Yen, 2019) evaluated laminectomy with additional facet denervation versus laminectomy alone and found no significant between-group differences at 6, 12, or 24 weeks postoperatively, thus concluding that routine intraoperative denervation cannot provide superior outcomes (Faqeeh and Yen, 2019). Our findings differ in that we observed higher early well-being in the RFA cohort; importantly, however, our study focused on a time window that is typically not assessed in surgical trials. One plausible explanation is that any association of RFA with recovery may be concentrated in the ultra-early postoperative phase (days to weeks), whereas longer-term disability outcomes may converge over time. If assessments begin at 6 weeks—as in the RCT—differences confined to the immediate postoperative phase may not be detected. The present feasibility study therefore suggests early between-group separation but does not establish durability or causality. Because medial branch RFA is time-limited due to nerve regeneration, any potential benefit may primarily influence the early convalescent period, whereas longer-term disability may depend more on the adequacy of decompression and underlying degenerative progression. Accordingly, future work should combine high-frequency early outcomes with conventional longer-term endpoints (ODI, pain, quality of life, and re-intervention) at 3–12 months to determine whether early trajectory differences translate into sustained clinical benefit.

Several factors further explain this discrepancy. First, stenosis populations exhibit heterogeneous facet pathology; trials that do not strictly select for facet-mediated pain may dilute the treatment effect (Cohen et al., 2020). Second, the effects of medial branch RFA are time-limited because the nerves regenerate (Smuck et al., 2012), which could help explain why an early signal would not necessarily persist at later follow-up. Third, while the magnitude of separation in our recovery trajectories is notable, our study was not blinded and expectation or reporting bias cannot be excluded. Finally, baseline axial low back pain intensity was not captured using a dedicated quantitative scale, so the extent to which pain burden influenced treatment selection or response cannot be determined.

### 4.3 Early recovery trajectory and high-frequency monitoring

The use of high-frequency, repeated patient-reported well-being during the early postoperative period allowed us to characterize recovery trajectories rather than relying on isolated clinic time points. Standard intermittent follow-up fails to capture the velocity of convalescence, often missing clinically relevant improvements in the "time-to-wellness." Our use of AUC metrics and trajectory modeling revealed that the RFA cohort exhibited not only higher well-being scores but also significantly faster improvement rates during the first 24 postoperative days. This approach illustrates the potential utility of ecological momentary assessment (EMA)-style monitoring in spine surgery for detecting transient early treatment associations that static time points may miss (Jaensson et al., 2017). Early symptom trajectories may also have prognostic relevance; delayed acute pain resolution has been linked to inferior long-term disability (Sato et al., 2024). However, in our study, any implications for mobilization, pain chronification, or downstream functional recovery remain hypothesis-generating because these constructs were not directly measured.

### 4.4 Feasibility and clinical implications of adding RFA

From a technical standpoint, the integration of adjunct RFA into standard decompression workflows is highly feasible. The medial branch nerve is anatomically accessible during posterior exposure, allowing for denervation under direct visualization as previously described (Lau et al., 2004). In our cohort, the addition of RFA extended operative time by approximately 20 min per level—an acceptable trade-off for a procedure intended to improve the early postoperative experience. Clinically, this technique may be relevant for selected stenosis patients with coexisting facet-mediated pain features who continue to be at risk of residual axial pain despite successful neural decompression (Ogon et al., 2022; Kim et al., 2013). Unlike fusion, which addresses back pain at the cost of motion loss and increased morbidity (Ghogawala et al., 2016), RFA targets the nociceptive generator without altering spinal biomechanics. Whether adjunct RFA influences opioid exposure, mobilization, rehabilitation milestones, or the need for additional fusion was not assessed here and should be addressed in future studies. Because the effect of denervation is time-limited (Smuck et al., 2012), adjunct RFA should presently be viewed as a hypothesis-generating perioperative strategy rather than a substitute for structural stabilization when instability is present.

### 4.5 Limitations and future directions

We acknowledge several limitations inherent to this feasibility study. First, as a non-randomized matched cohort with a small sample size (n = 40), the analysis is vulnerable to residual confounding and does not support causal inference. In particular, allocation to adjunct RFA was based on a predefined clinical-radiological phenotype, so confounding by indication and phenotype-specific responsiveness cannot be excluded despite matching. Second, the absence of blinding introduces potential expectation and reporting bias. Third, the principal early-recovery measure was a single-item well-being scale used as an exploratory pragmatic digital monitoring tool rather than a formally validated standalone postoperative recovery instrument for this population. Although it was useful for capturing recovery dynamics, its measurement validity and susceptibility to reporting bias require acknowledgement. Fourth, the main between-group differences were observed only during the first 24 postoperative days, whereas ODI and PHQ-9 at later follow-up were similar; therefore, the present findings cannot be extrapolated to mid- or long-term outcomes. Fifth, baseline axial low back pain intensity was not captured using a dedicated quantitative scale, limiting assessment of how symptom burden may have influenced treatment selection and response. These data provide the rationale for a definitive randomized controlled trial comparing decompression plus RFA versus decompression alone. Future study designs should prioritize blinding, explicit quantification of axial back pain, stratification by facet pathology, and integration of granular early recovery metrics (AUC, time-to-wellness) with standard longer-term outcomes and resource-utilization data to fully characterize the clinical value and cost-effectiveness of this adjunct procedure.

## 5 Conclusion

In this prospective matched feasibility study, adjunct facet RFA was associated with better early postoperative well-being trajectories during the first 24 days after lumbar decompression. This association did not extend to significant between-group differences in ODI or PHQ-9 at later follow-up and should not be interpreted as proof of causal or durable benefit. The findings highlight both the potential value of granular patient-reported monitoring in spine surgery and the limitations of static follow-up intervals for capturing early recovery. A definitive blinded randomized trial is warranted to determine whether this early signal persists in rigorously selected patients.

## Consent to participate

Informed consent for clinical documentation and electronic patient-reported outcome measures (PROMs) as part of routine clinical practice was obtained for eligible patients. The study was conducted in accordance with the ethical principles outlined in the Declaration of Helsinki. Approval for the scientific evaluation of those data was obtained from the Ethics Committee of the Medical Faculty, Heidelberg University (approval number: S-096/2025).

## Consent to participate

Analysis of these routinely collected clinical data was covered under the institutional neurosurgical registry ethics approval (Ethics Committee of the Medical Faculty, Heidelberg University; approval number: S-096/2025), and thus separate informed consent for scientific evaluation was not required.

## Note

Funding acquisition is not listed because the manuscript states that the research received no specific grant from public, commercial or not-for-profit funding agencies.

## Consent for publication

No individual person's data were included in this study.

## Data material availability

The datasets generated during and/or analyzed during the current study are available from the corresponding author on reasonable request.

## Ethics approval

This study was approved by the Institutional Review Board of Heidelberg University (approval no. S-607/2023 and S-096/2025).

## Authors’ contributions

All authors contributed to the study conception and design. Data collection and analysis were performed by Pavlina Lenga, Sandro Krieg and Martin Dugas. The first draft of the manuscript was written by Pavlina Lenga. Sandro Krieg, Martin Dugas, Robin Fleige, Max Christian Blumenstock, Matthias Ganzinger and Sebastian Ille commented on previous versions of the manuscript. All authors read and approved the final manuscript.

## Funding

This research received no specific grant from any funding agency in the public, commercial, or not-for-profit sectors.

## Declaration of competing interests

The author(s) declared no potential conflicts of interest with respect to the research, authorship, and/or publication of this article.
