# Supplementary material for: Adjunct facet radiofrequency ablation accelerates early recovery trajectories after lumbar Decompression: A prospective matched feasibility study
Source: Brain Spine. 2026 Sep 5;6:106057. doi: 10.1016/j.bas.2026.106057 (PMC13602701; doi:10.1016/j.bas.2026.106057)
Supplement: Supplementary file 1 [file mmc1.docx]

**Supplementary Table S1. Surgical characteristics (estimated).**

| **Group** | **Levels** | **n** | **Segments** | **Total op time (min)** | **Decompression time (min)** | **RFA time (min)** |
| --- | --- | --- | --- | --- | --- | --- |
| RFA+Decompression | 1 | 10 | L4/5 | 81.5 ± 1.1 | 61.9 ± 1.2 | 21.8 ± 2.1 |
| RFA+Decompression | 2 | 10 | L4/5+L5/S1 | 162.3 ± 1.2 | 122.1 ± 2.8 | 42.8 ± 1.9 |
| Decompression only | 1 | 10 | L4/5 | 62.5 ± 2.8 | 61.2 ± 3.2 | 0.0 ± 0.0 |
| Decompression only | 2 | 10 | L4/5+L5/S1 | 122.1 ± 1.2 | 121.8 ± 2.5 | 0.0 ± 0.0 |

**Supplementary Table S2. Subgroup analyses by operated levels (paired).**

| **Levels** | **Outcome** | **RFA mean** | **Control mean** | **Diff** | **95% CI** | **p (paired t)** |
| --- | --- | --- | --- | --- | --- | --- |
| 1 | Well-being mean | 83.2 | 45.5 | 37.7 | 27.6 to 47.7 | <0.001 |
| 1 | Well-being last | 84.5 | 42.0 | 42.5 | 32.2 to 52.8 | <0.001 |
| 1 | ODI postop | 22.5 | 33.9 | -11.4 | -28.4 to 5.7 | 0.154 |
| 1 | ODI improvement | 15.4 | 20.1 | -4.6 | -28.8 to 19.6 | 0.656 |
| 2 | Well-being mean | 69.0 | 47.8 | 21.2 | 12.7 to 29.8 | <0.001 |
| 2 | Well-being last | 75.0 | 46.6 | 28.4 | 8.4 to 48.4 | 0.011 |
| 2 | ODI postop | 32.5 | 32.6 | -0.1 | -25.0 to 24.8 | 0.991 |
| 2 | ODI improvement | 21.9 | 15.9 | 6.0 | -55.0 to 67.0 | 0.713 |
